# An update on pediatric traumatic brain injury

**DOI:** 10.1007/s00381-023-06173-y

**Published:** 2023-10-06

**Authors:** Anthony Figaji

**Affiliations:** Division of Neurosurgery and Neurosciences Institute, University of Cape Town, Cape Town, South Africa

**Keywords:** Traumatic brain injury, Children, Pediatric, Intracranial pressure

## Abstract

**Introduction:**

Traumatic brain injury (TBI) remains the commonest neurological and neurosurgical cause of death and survivor disability among children and young adults. This review summarizes some of the important recent publications that have added to our understanding of the condition and advanced clinical practice.

**Methods:**

Targeted review of the literature on various aspects of paediatric TBI over the last 5 years.

**Results:**

Recent literature has provided new insights into the burden of paediatric TBI and patient outcome across geographical divides and the severity spectrum. Although CT scans remain a standard, rapid sequence MRI without sedation has been increasingly used in the frontline. Advanced MRI sequences are also being used to better understand pathology and to improve prognostication. Various initiatives in paediatric and adult TBI have contributed regionally and internationally to harmonising research efforts in mild and severe TBI. Emerging data on advanced brain monitoring from paediatric studies and extrapolated from adult studies continues to slowly advance our understanding of its role. There has been growing interest in non-invasive monitoring, although the clinical applications remain somewhat unclear. Contributions of the first large scale comparative effectiveness trial have advanced knowledge, especially for the use of hyperosmolar therapies and cerebrospinal fluid drainage in severe paediatric TBI. Finally, the growth of large and even global networks is a welcome development that addresses the limitations of small sample size and generalizability typical of single-centre studies.

**Conclusion:**

Publications in recent years have contributed iteratively to progress in understanding paediatric TBI and how best to manage patients.

## Introduction

Traumatic brain injury (TBI) remains the commonest neurological and neurosurgical cause of death and survivor disability among children and young adults across the world. The Global Burden of Disease 2016 study estimated that there were about 27 million new cases of TBI across the world, and just under 1 million cases of spinal cord injury that year [1]. The number of TBI cases may actually be much larger, in the region of 50–60 million cases/year [2]. To put this into perspective, there were around 10 million new cases of tuberculosis in 2015 [3]. Moreover, it is likely that the incidence of TBI is under-reported because it is not a registrable condition and there is little organized data collection for TBI across the world, especially in LMICs. Based on the available data, it is estimated that individuals living with a TBI-related disability have a global age-standardized prevalence of 759 per 100,000 people, corresponding to 55 million individuals with TBI [1]. Still, there are surprisingly few large international initiatives in the field, and funding for organized research remains inexplicably small, especially given the potential for research-directed clinical care to make a significant difference in outcomes. For historical reasons, the funding for organized studies in pediatric TBI is dwarfed by funding for infectious, cardiovascular, and neurodegenerative diseases.

This current review does not aim to comprehensively cover the field of pediatric TBI—several past reviews have adequately provided an overview of pediatric TBI clinical care and research. Rather, this review aims to provide an update on some of the relevant work on the subject in the past 5 years and suggest promising areas for future research.

## New insights in the burden of pediatric TBI and patient outcome

When evaluating the epidemiology and outcomes of pediatric TBI in published literature, it is important to consider the age range in the studies because this affects how we should interpret the data. Many centers in the USA report data for children *and* adolescents, up to age 19 or even 21 [4, 5], whereas other institutions variably have a lower age cutoff, around 13–14 years old [6]. This is important because the mechanisms of injury differ across the age range, and the underlying physiology of a young child is very different from that of an adolescent, which progressively approximates adult physiology [7]. How one assesses developmental outcomes also differs substantially across the age range and requires the use of multiple tools [8]. Sex differences may also influence the child’s response to injury, their response to interventions, and their overall recovery [9]. Finally, *where* the study was conducted also matters. For example, the predominant mechanisms of injury vary across the world, mostly influenced by the socio-economic conditions of a region. Social determinants of outcome after pediatric TBI may have a significant impact on outcome, especially in the rehabilitation phase, reflecting the health disparities within every community [10]. Furthermore, epidemiology within regions may also change over time, due to changes in societal organization and pressures, such as increased motor vehicle accidents associated with rapid urbanization in LMICs. In the USA, other changes, such as an increase in suicide among children [11] and increasing incidence of firearm injuries, will affect trauma statistics [12].

Although traditionally, the focus on TBI care was largely centered on patients with severe TBI, in the last decade, this has shifted somewhat to mild TBI and sports-related concussion. Either way, functional outcomes matter at both ends of the spectrum and require long-term evaluation for full understanding. Tracking measures of executive functioning have revealed that even patients with mild TBI may demonstrate poorer function at follow-up than controls, and children with severe TBI may continue to deteriorate even after plateauing for more than a year post-injury [13, 14].

Young children are particularly vulnerable to TBI-related developmental delays [14]. The long-term risks for these children are not inconsiderable, not only for learning disabilities but also for the development of psychiatric disorders [15] and possibly even criminality [16]. When prognosticating and evaluating the effectiveness of interventions, it is important to remember that there may be racial/ethnic differences in outcome. Much of this difference may be a proxy for socio-economic differences in communities, but there also may be regional and ethnic differences in cellular responses to injury and disease [17]. Increasingly, the influence of population or individual genetics on outcome after TBI has been recognized [18, 19].

Increasingly, in the era of large datasets and machine-learning approaches, outcome prediction models have improved over time, with areas under the curve as high as 0.9 [20, 21]; however, the degree to which these will be used in clinical practice to prognosticate for individual patients remains unclear. Still, it is likely that these methods will continue to improve and be increasingly used clinically in time.

Non-accidental injury (NAI) or abusive head trauma (AHT) continues to be a major societal cause of TBI and a leading cause of death and disability in the very young. Most children who are admitted with a NAI have sustained a TBI, and around 70% are under the age of one [22]. An analysis of outcomes from a national database of 10,965 children with TBI found that children with AHT had higher mortality compared to TBI due to motor vehicle collision after adjustment for relevant confounders [23].

In terms of post-traumatic epilepsy outcomes, a recent systematic review found an overall incidence of 10% after pediatric TBI, with significant predictors, unsurprisingly, being severe TBI classification, intracranial hemorrhage, and the occurrence of early seizures [24]. Elsamadicy et al*.* found similar results after analysis of data from a large national database [5]. When one examines patients in greater depth, post-traumatic epilepsy associates also with increased measures of acute care measures: increased ICP, increased pressure reactivity index, worse findings on head CT, decreased heart rate variability, and the presence of epileptiform discharges and abnormal sleep spindles [25].

Finally, it is important also to remember the psychological and financial burden on families of children who have suffered a TBI. Extrapolating results from a survey, Nelson et al*.* estimated that pediatric TBI was associated with more than 670,000 lost workdays annually over 12 months post-injury in the USA, translating into more than US $150 million in lost productivity [26].

## Imaging

CT-based imaging remains the most common form of emergency cranial imaging for pediatric TBI, despite the growing concerns about radiation risks. This remains so largely due to its wide availability, its speed, and its sensitivity to detect clinically relevant pathology. Various rules, such as from the PECARN group, have been introduced to guide clinical decisions about which children with mild TBI should undergo imaging [27]. However, MRI is being used with increasing frequency, especially in the USA. Data from the ADAPT trial showed that MRI was used much more frequently at the US sites compared to the international ones (94% of US sites versus 44% of international sites performed MRI in at least 70% of children with severe TBI, and 40% of sites reported obtaining MRI in more than 95% of these cases [28]. In particular, rapid sequence MRI without sedation is becoming increasingly popular to avoid CT-radiation. Lindberg et al*.* [29] reported their experience with a fast MRI protocol incorporating multiple sequences: their median time to completion was 6 min in unsedated patients, compared to a 1 min completion time with head CT. MRI missed pathology in 8 out of 111 patients, including 6 isolated fractures and 2 subarachnoid hemorrhage, all arguably not clinically significant. Shope et al*.* published similar findings: their protocol took 7 min to complete with no sedation used; in their study, MRI outperformed CT in all pathologies other than for skull fractures, for which MRI had a low sensitivity [30].

In research, the ENIGMA group has established a network of centers engaged with the study of advanced MRI sequences to better understand pediatric moderate and severe TBI and discover new imaging biomarkers for prognostication [31]. It is important to remember that the characteristics of TBI imaging are different in children than adults: in addition to the greater proportion of diffuse injury in children, just because basal cisterns are open does not mean that ICP is not elevated [32], and the manifestations of pathological ICP on head CT may be different to that of adults [33].

## Guidelines and consensus documents

Currently, there are no internationally constituted and adopted guidelines for pediatric TBI care. The most widely cited is the Brain Trauma Foundation–supported guidelines for the Management of Pediatric Severe Traumatic Brain Injury [34], which has some limitations in generalization and practicality based on regional authorship constitution and strict evidence-based approach [35], but remains an important contribution to the existing guidance on the topic. Being restricted by the evidence-based guideline process, the document could make few strong recommendations because the evidence base was weak: there were no level I recommendations, only 3 level II recommendations (two of which were negative recommendations of what *not* to do), and the rest were level III, which in the old terminology were considered “options”. The level II recommendations were as follows: (1) Bolus hypertonic saline (3%) is recommended for patients with intracranial hypertension (for ICP control), (2) prophylactic moderate (32 to 33 °C) hypothermia *is not* recommended over normothermia to improve overall outcomes, and (3) use of an immune-modulating diet *is not* recommended (to improve overall outcomes) [34]. A useful new addition to their approach was the development of a proposed algorithm for first and second tier therapies, creating several pathways depending on circumstance and the monitoring approach used: pathways based on a herniation concern, ICP monitoring, CPP-targeting, and brain oxygenation monitoring-directed [36].

In 2018, the US–based Centers for Disease and Prevention published a guideline for the diagnosis and management of children with mild TBI [37]. This document was a North American initiative to develop guidelines for childhood mild TBI in line with that which had previously been developed for adult TBI. The guidelines recognized the current interest in biomarker research but agreed that there is insufficient evidence for the use of biomarkers in pediatric TBI outside a research setting. There was no single recommendation for tools used in assessment and prognosis of mild TBI in children, citing the need for age-appropriate rating scales and awareness of the variability in recovery across patients. The paper repeats widely held recommendations for some form of cognitive rest after mild TBI with gradual return to activity titrated against symptoms, also recognizing that return to exercise may be beneficial in some patients with prolonged symptoms as long as this did not exacerbate those symptoms.

Recently, the Concussion in Sport Group updated recommendations for a tool used in assessment of sports-related concussion in the subacute period, the Sports Concussion Office Assessment (SCOAT6) [38].

Of parallel interest, because spinal cord injury may occur combined with TBI, there is a recently published Delphi consensus for the medical management of spinal cord injury in children [39].

In the adult literature, the Brain Trauma Foundation published their fourth edition of their guidelines in 2017 [40], with a commitment to updating this as a living document as new research is published. They this did recently for decompressive craniectomy [41] to update their recommendation after the RESCUE-ICP trial was published. A new group (SIBICC) took a different approach, recognizing the ongoing need for practical recommendations to clinicians which formal guidelines often cannot do due to lack of good quality data. The work produced a series of consensus statements developed from a broadly comprised group of neurosurgeons, emergency care physicians, and intensivists. Using a consensus-based approach, they first published two management algorithms, one for ICP-only monitored patients [42] and one for patients managed with both ICP and brain oxygenation monitoring [43]. Both sets of recommendations were based on a system of interventions divided into tiers, with inter-tier considerations and recommendations for neuroworsening. In the ICP management algorithm, autoregulation was incorporated for the first time, and they produced heatmaps for graded approaches to ICP monitor removal and sedation holidays. For patients managed with both ICP and brain oxygen monitoring, they produced protocols for patients with intracranial hypertension where brain oxygen was normal and when there was brain hypoxia. The group later went on to develop recommendations for prognostication and goals of care decisions [44]. Finally, there is a current process to develop a new set of recommendations for penetrating TBI [45].

## Brain monitoring

### Autoregulation

Pressure autoregulation is the ability of cerebral arterioles to vary their diameter in response to changes in blood pressure, thereby maintaining a reasonably constant cerebral blood flow. The change in vascular diameter also affects cerebral blood volume, and therefore, ICP if intracranial compliance is reduced. In patients with TBI, autoregulatory capacity may be weakened or even absent, and this is unpredictable without formal monitoring. As a consequence, the interaction between blood pressure and intracranial dynamics may change. Therefore, variations in autoregulatory capacity have implications for the relationship between blood pressure, cerebral blood flow, and ICP. Following on from this, it has important implications for what happens to intracranial dynamics when cerebral perfusion pressure targets are pursued using inotropes and fluid boluses. The SIBICC consensus discussed above is one of the first to incorporate autoregulatory status as a consideration in its algorithm for ICP-directed care in adults [42]. Less has been known about how autoregulatory status should direct clinical care in pediatric TBI, if at all, because although several studies have examined autoregulatory capacity in children, demonstrating its variability in severe TBI and association with outcome [46–48], cohort sizes have been small. Recently, Smith et al*.* published the largest series to date (196 children) using the pressure-reactivity index, a moving correlation co-efficient between blood pressure and ICP (as a proxy for cerebral blood volume) [49]. Their results showed that impaired autoregulation was as strongly associated with mortality as high ICP and that the association was independent of both ICP and admission Glasgow Coma Score, suggesting that it is not merely a proxy for severity of injury. Given that the status of autoregulation has implications for what happens to intracranial dynamics when blood pressure is manipulated, it is plausible that it should be taken into account when making decisions about CPP management.

### ICP monitoring

The BEST-TRIP trial in adult TBI failed to demonstrate a benefit of a specific protocol for ICP monitoring in a resource-constrained environment where ICP monitoring had not previously been available [50]. For many reasons, the results have not thought to be generalizable to more established critical care environments or where different treatment protocols are employed. Therefore, ICP monitoring remains a cornerstone in adult [40] and pediatric [34] guidelines, although the precise indications for initiating ICP monitoring remain unclear. A UK survey of practice in pediatric ICUs showed little consistency in what indications were used for ICP monitoring and what cerebral perfusion pressures were targeted [51]. A more recent large observational study in adult ICUs in a high-income setting appears to favor ICP monitoring [52], and new management algorithms have been published [42]. In a comparative effectiveness study of adult penetrating TBI, mortality was 31% versus 41%, respectively, in patients who received monitoring compared to those who did not [53]. It is likely though that intracranial hypertension in TBI is complex and variable between patients and therefore cannot be approached as a simple protocol targeting a single number [54].

### Brain oxygen monitoring

In adult TBI, a phase II trial of ICP-directed versus ICP *plus* brain oxygen-directed care favored the latter [55], and phase III studies are now underway. In pediatric TBI, studies of brain tissue oxygenation monitoring have been smaller and all observational; however, collectively, they suggest that brain tissue oxygen monitoring detects cerebral hypoxic episodes that would have been otherwise unnoticed, that these episodes are associated with poor outcomes, and that treatment directed by monitoring may benefit patient care [56–60]. It is still uncommonly used though; in North America, 90% of surveyed pediatric ICUs used ICP monitoring, but just under 20% used brain tissue oxygen monitoring [61].

### Non-invasive brain monitoring

Recent interest in non-invasive methods for measuring ICP and CPP has grown, especially for using them as screening or monitoring tools for patients in whom the indication for invasive monitoring is not met. Optic nerve sheath ultrasound in particular has seen a surge in interest, with varied degrees of correlation with measured ICP being reported [62–65]. There remain some concerns about inter-observer agreement, measurement precision, and observer bias [66, 67], but it is likely that interest will remain high and techniques will improve. Similarly, there have been concerns about the sensitivity and specificity of transcranial Doppler-derived indices for predicting ICP [68], but further innovations and experience appear to be improving its predictive power, especially for CPP [69, 70], and overall usage for different conditions in an intensive care setting has increased, albeit being used for various purposes [71]. In a small cohort of children, Fanelli et al. [72] collected data on invasive ICP and arterial blood pressure recordings, along with TCD-based determination of cerebral blood flow velocity waveforms. From the automated pipeline they developed using the waveforms, their non-invasive estimates of ICP had a receiver operating characteristic curve of 0.83, with a sensitivity of 71% and specificity of 86% for ICP greater than 15 mmHg. These tests are promising; however, they do require training and remain difficult to conduct continuously. A North American survey of pediatric intensive care units revealed that 8% measured optic nerve sheath diameter, 33% used pupillometry, 87% used near-infrared spectroscopy, 100% used continuous EEG, and 100% used intermittent TCD (10% continuous) [61].

### Other monitoring

Continuous EEG is being increasingly used in pediatric ICUs and is available in most centers [51]. It is used to detect subclinical seizures, to monitor sedation levels, and to prognosticate. The detection of subclinical seizures has particular value because seizures may increase ICP and increase metabolic demand. It is worth noting, however, that scalp EEG may not detect all subclinical seizures: Appavu et al*.* demonstrated this in using intracranial electroencephalography along with scalp electrodes [73].

Other monitoring tools used in severe TBI management such as microdialysis are still largely considered only at research centers, in part because of the expense and infrastructure required; however, there are many aspects of TBI that can be interrogated at the bedside through its use [74, 75].

Interest has also grown in measures that may predict subtle changes in function that may correlate with brain injury severity and prognosis in mild TBI and concussion. One such method is eye tracking, for which several new devices have been brought to market [76–79] but arguably still need widespread evaluation before adoption as a standardized technique. For example, in a cohort of 56 children with concussion, Zahid et al. found that eye tracking metrics correlated with symptoms and detected accommodative and convergence abnormalities [79].

## Therapies

### Hyperosmolar therapy

For various reasons, there has been a trend in pediatric circles away from mannitol and towards hypertonic saline (HTS) as a hyperosmolar agent in the treatment of intracranial hypertension in children. The findings of the recent ADAPT study were in keeping with this: the comparative effectiveness study examined 518 enrolled children with severe TBI and ICP monitoring who received bolus hyperosmolar therapy [80]. HTS was given around 3 times more often than mannitol and was associated with both lower ICP and higher CPP, compared to mannitol which was associated only with higher CPP. During ICP crises, HTS fared better than mannitol. CENTER-TBI studied a similar number of adults with TBI who received hyperosmolar therapy [81]. Again, HTS was more commonly used but not by as much of a difference as was the case in ADAPT. There was no difference between patients who received HTS or mannitol, and the center in which the patients were treated was a stronger predictor of which therapies patients received than their injury characteristics.

A recent randomized controlled trial across several centers in France did not show a benefit of HTS continuous infusion against standard of care [82]; however, this was not targeted as a treatment for intracranial hypertension, which is how most centers use the therapy. The standard of care group received hyperosmolar therapy anyway if it was indicated for intracranial hypertension. Not all patients would have had intracranial hypertension; therefore, it can be argued that the potential benefits of hyperosmolar therapy in patients with clearly documented intracranial hypertension may have been different and that there is little value of HTS without intracranial hypertension.

### CSF drainage

Placement of an external ventricular drain (EVD) is a standard and common procedure in TBI care because it can be used to monitor ICP and reduce ICP by CSF drainage. As such, it is often included as an early tier approach in TBI management. However, this has never been subject to a controlled trial. On the negative side, not all patients require CSF withdrawal, and the use of an EVD is not without complications. First, accurate placement of an EVD is not straightforward when the ventricles are small, and multiple attempts are ill-advised in an already swollen brain. The risk of hemorrhage is higher than with an intraparenchymally placed monitor, as are the infection risks, given that it is a fluid-coupled device. As such, wide practice variations exist. Typically, EVDs are used more commonly in the USA than in European centers, but significant differences also exist across European countries [83]. A recent important comparative effectiveness study from the ADAPT group enrolled 1000 children with ICP monitoring for severe TBI, 314 of whom received CSF drainage [84]. Outcomes were slightly worse in the CSF drainage group for mortality and functional score (non-significantly), although the ICP was lower. This may reflect a selection bias as there were slight differences between groups. When propensity matching was performed, there was no difference between the two groups; however, the numbers in the propensity matched groups were small. Although the adult guidelines still support placement of an EVD early in the course, there are a few concerns from the literature. The study by Griesedale et al*.* [85] found that CSF drainage was associated with increased mortality in 93 adults compared with 73 without CSF drainage. The findings from Bales et al*.* [86] were similar: higher mortality and worse functional outcomes occurred in the CSF drainage group. It is also possible that, while CSF drainage may benefit patients with intracranial hypertension, the *routine* use of EVDs may not benefit patient care. Further clarity can be obtained only if the patient groups are similar, as in a RCT.

### Decompressive craniectomy

There have been no randomized trials of decompressive craniectomy in pediatric TBI, other than the small pilot study of Taylor et al*.* more than 20 years ago in which the surgical procedure was nothing like that which is currently used [87]. The adult trials of DECRA [88], RESCUE-ICP [89], and most recently RESCUE-ASDH [90] have shed some light on the topic, but in many ways do not clarify the way forward for children. The procedure remains fairly common in pediatric TBI—in the ADAPT study of 1000 children with ICP monitoring for severe TBI across more than 50 centers; mostly in the USA and the UK, around 20% of patients underwent decompressive craniectomy [84]. Although there have been guideline updates for decompressive craniectomy in adults as well as consensus statements, recommendations for children remain unclear. Similarly, there are less data on cranioplasty following craniectomy in children. Although a recent consensus document addresses the topic in both adults and children [91], there remains much uncertainty, especially in young children where bone resorption is higher and the use of synthetic material is problematic because the cranium is still growing. There is a current prospective study in Europe recruiting data on cranioplasty outcomes for children [92].

### Tracheostomy

The use of tracheostomy after TBI is variable across different centers. Recent reports examined the outcomes. Sheehan et al*.* examined early and late tracheostomy outcomes in 127 children with TBI in a national database; their results suggested the same overall outcomes in the two groups, but a decrease in ICU stay and ventilator days in the early tracheostomy group [93]. Salik et al*.* performed a similar analysis in a different national database that queried 1956 children who underwent tracheostomy for TBI care. Patients who underwent tracheostomy were typically older and had more severe disease. After propensity matching, early tracheostomy was similarly associated with reduced ICU stay and ventilator days compared to late tracheostomy [94]. Finally, McLaughlin et al*.*, in 121 propensity matched pairs, also found that early tracheostomy is associated with shorter hospital stay and fewer complications [95].

## Global pediatric TBI research

There are many reasons why a global approach would benefit pediatric TBI research. First, most centers treat a relatively small volume of patients; large patient cohorts are usually needed to adequately power studies. Second, not all centers are the same: mechanism of disease, case mix, admission policies, acute treatment protocols, and rehabilitation capacity all vary across centers. Therefore, results from individual centers may not generalize to centers where circumstances are different [96]. Third, most published work emanates from high income centers but the vast majority of cases occur in low- and middle-income countries [97]. Therefore, guidelines developed from published data may not deliver the same outcomes in places where the condition predominates. To address this, there have been recent attempts to regionally adapt guidelines to resource constraints [98].

In recent years, several networked groups have formed to address issues directly or indirectly pertinent to TBI care, but these remain constrained in their ability to address the above limitations. For example, several specialty groups in the USA have developed collaborative networks to increase statistical power and generalizability, including the PECARN group [27] and Pediatric Neurocritical Care Research group (PNCRG) [61]. However, their data are generated from, and their work is focused on, US sites. Data and application may be very different elsewhere. The ADAPT trial [99] was a novel comparative effectiveness trial that created an impressive collaboration across more than 50 centers that prospectively collected observational data. However, ICP monitoring was a requirement, and the sites, with the exception of two, were all based in high-income countries, predominantly the USA and the UK. CENTER-TBI [100] and TRACK TBI [101] were impressive achievements with substantial funding, but again only addressed care in predominantly high-income countries and only in adults. Significant funding and appropriate global networks are urgently needed for the study of pediatric TBI, especially where the condition is most common and for which relevant results would be most impactful. In doing so, we could increase statistical power, improve the generalizability of data, and positively influence the care of the largest number of children across the child suffering from one of the most common causes of avoidable death and disability.
